# Two Efficient Applications in Hemorrhoids

**Published:** 1912-09

**Authors:** 


Two Efficient Applications in Hemorrhoids.—W. J. Robinson, *N. Y. Med. Jour.*, July 6, 1912.

I.

| | | |
|---|---|---|
| ℞ | Pulveris gallæ, | ʒi; |
| | Zinci oxidi, | ʒi; |
| | Hydrargyri chloridi mitis, | ʒss; |
| | Bismuthi subnitratis, | ʒi; |
| | Cocainæ hpdrochloridi, | gr. v; |
| | Unguenti aquæ rosæ, | ℥i. |
| M. | Fiat unguentum. | |

II.

| | | |
|---|---|---|
| ℞ | Pulveris gallæ, | grs. v; |
| | Zinci oxidi, | grs. v; |
| | Morphinæ sulphatis, | gr. ¼; |
| | Cocainæ hydrochloridi, | gr. i; |
| | Atropinæ sulphatis, | gr. 1/60; |
| | Olei theobromæ, | grs. xxv. |
| M. | Fiat suppositorium No. 1. Da tales doses No. 24. | |

In external piles the ointment is used, well smeared in, within and around the anus, and protected with a piece of cotton partially inserted therein. In internal piles the suppositories are used. Sometimes both are required. The suppositories are used once or twice, the ointment three times a day.

